# Hand Sanitizers: A Review on Formulation Aspects, Adverse Effects, and Regulations

**DOI:** 10.3390/ijerph17093326

**Published:** 2020-05-11

**Authors:** Jane Lee Jia Jing, Thong Pei Yi, Rajendran J. C. Bose, Jason R. McCarthy, Nagendran Tharmalingam, Thiagarajan Madheswaran

**Affiliations:** 1 School of Pharmacy, International Medical University, No. 126 Jalan Jalil Perkasa 19, Bukit Jalil, Kuala Lumpur 57000, Malaysia; JANE.LEEJIA@student.imu.edu.my (J.L.J.J.); THONG.PEIYI@student.imu.edu.my (T.P.Y.); 2 Masonic Medical Research Institute, 2150 Bleecker St, Utica, NY 13501, USA; jcb.bhuvana@gmail.com (R.J.C.B.); jmccarthy@mmri.edu (J.R.M.); 3 Infectious Diseases Division, Warren Alpert Medical School of Brown University, Rhode Island Hospital, Providence, RI 02903, USA; nagendran_tharmaligam@brown.edu; 4 Department of Pharmaceutical Technology, International Medical University, No. 126 Jalan Jalil Perkasa 19, Bukit Jalil, Kuala Lumpur 57000, Malaysia

**Keywords:** hand sanitizer, hand disinfectants, infection control

## Abstract

Hand hygiene is of utmost importance as it may be contaminated easily from direct contact with airborne microorganism droplets from coughs and sneezes. Particularly in situations like pandemic outbreak, it is crucial to interrupt the transmission chain of the virus by the practice of proper hand sanitization. It can be achieved with contact isolation and strict infection control tool like maintaining good hand hygiene in hospital settings and in public. The success of the hand sanitization solely depends on the use of effective hand disinfecting agents formulated in various types and forms such as antimicrobial soaps, water-based or alcohol-based hand sanitizer, with the latter being widely used in hospital settings. To date, most of the effective hand sanitizer products are alcohol-based formulations containing 62%–95% of alcohol as it can denature the proteins of microbes and the ability to inactivate viruses. This systematic review correlated with the data available in Pubmed, and it will investigate the range of available hand sanitizers and their effectiveness as well as the formulation aspects, adverse effects, and recommendations to enhance the formulation efficiency and safety. Further, this article highlights the efficacy of alcohol-based hand sanitizer against the coronavirus.

## 1. Introduction

The emergence of the COVID-19 (Coronavirus Disease-2019) pandemic has risen to be a significant global public health concern and led to extensive use of hand disinfectants given its contagious nature. There was a total of 3.8 million reported cases affecting over 200 countries worldwide as of 7 May 2020 [1,2]. COVID-19 is an infectious disease caused by the severe acute respiratory syndrome coronavirus 2 (SARS-CoV-2), which can persist and remain infectious on surfaces for up to 9 days [3,4]. The recent study reveals that transmission of SARS-CoV-2 is possible in the form of aerosol and fomite, and the virus can remain viable and infectious in aerosols for hours and on surfaces up to days, depending on the inoculum shed [5]. Hence, it is crucial to interrupt the transmission chain of the virus through contact isolation and strict infection control tools [6]. Following face masks, appropriate hand hygiene is of utmost importance as hands may be contaminated from direct contact with patients’ respiratory droplets from coughs and sneezes or indirect contact via surfaces, which may then facilitate the transmission and spreading of the disease [7,8,9]. The 2003 severe acute respiratory syndrome (SARS) outbreak was caused by a novel human coronavirus (CoV) (SARS-CoV) that could survive on surfaces for 24 to 72 h [10]. The studies on SARS-CoV outbreak settings showed that providing efficient handwashing facilities reduced transmission [11].

**Figure 1:**
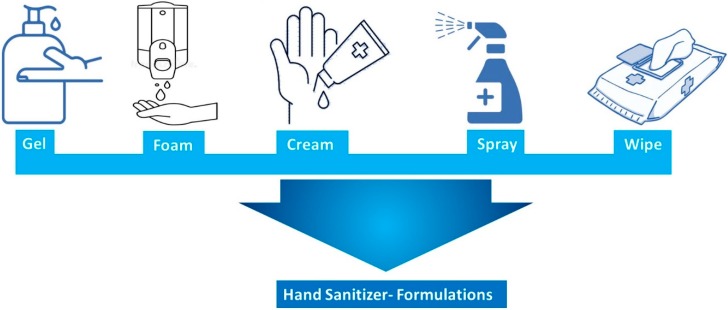
Various types of hand sanitizer dosage forms.

Given the dangers imposed by this disease, the Centre for Disease Control and Prevention (CDC), the United States has promoted and encouraged hand hygiene through handwashing or use of hand sanitizer [12]. Hand disinfectants are commercially available in various types and forms such as anti-microbial soaps, water-based or alcohol-based hand sanitizers, most often used in hospital settings. Different types of delivery systems are also formulated—for instance, rubs, foams, or wipes (Figure 1). The World Health Organisation (WHO) recommends alcohol-based hand sanitizer (ABHS) in line with the proven advantages of their rapid action and a broad spectrum of microbicidal activity offering protection against bacteria and viruses. However, the effectiveness against non-enveloped viruses is still debatable and questionable [7,13,14,15,16,17,18].

To date, most effective hand sanitizer products are alcohol-based formulations containing 62%–95% of alcohol as it is capable of denaturing the proteins of microbes and inactivating viruses [19,20]. There are a few challenges and concerns with regard to this formulation in terms of fire hazards and skin toxicity due to high alcohol content [21]. This systemic review aims to investigate the range of available hand sanitizers and their effectiveness against the human coronavirus as well as the formulation aspects, adverse effects, and recommendations to improve the formulation of current hand sanitizers.

## 2. Methods

This study was conducted according to the PRISMA recommendations [22]. We systematically reviewed the available literature in PubMed and Google Scholar, up to 2020. The search terms we used are hand sanitizers AND alcohol AND treatment AND handwashing AND virucide AND bactericide AND (cure OR failure OR mortality). A manual search was also performed. We set no year limit, and English is the only language we limit. The study selection based on effective treatment resulted in a potential eradication of pathogens. The data extracted from each study comprised the main characteristics of the study, such as the first author’s name, year, study design, and country. Out of many reports, we selected articles based on the hand disinfectant agents and their potential outcome suitable for the present viral pandemic. Data were extracted by two authors based on the screening of the titles and abstracts obtained from the PubMed and Google Scholar database. The other authors have checked the materials to fulfil the criteria for the work.

## 3. Results and Discussion

### 3.1. Types of Hand Sanitizer

**Figure 2:**
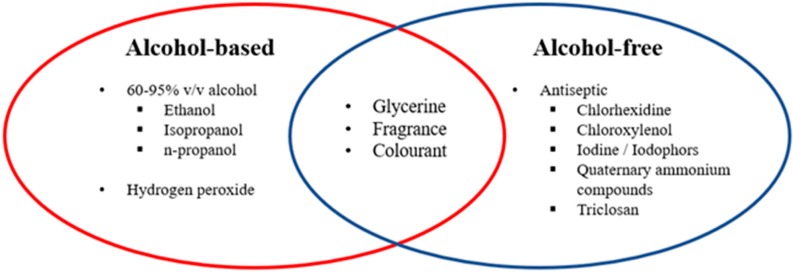
List of alcohol, non-alcohol compounds, and commonly used excipients in hand sanitizers.

Hand sanitizer can generally be categorized into two groups: alcohol-based or alcohol-free (Figure 2). An ABHS may contain one or more types of alcohol, with or without other excipients and humectants, to be applied on the hands to destroy microbes and temporarily suppress their growth [23]. ABHS can effectively and quickly reduce microbes covering a broad germicidal spectrum without the need for water or drying with towels. Nevertheless, there are a few shortcomings with the effectiveness of ABHS, such as its short-lived antimicrobial effect and weak activity against protozoa, some non-enveloped (non-lipophilic) viruses and bacterial spores [23].

On the other hand, the alcohol-free sanitizer makes use of chemicals with antiseptic properties to exert the antimicrobial effects. These chemicals have a different mode of action and function according to their chemical functional groups (Table 1) [24,25,26]. As they are non-flammable and often used at low concentrations, they are relatively safer to use among children as compared to ABHS.

ABHS is available in different dosage forms, namely gel, liquid and foam. As each type has its own characteristics, a study was conducted to understand the impact on sensory attributes that may affect user’s acceptance of the product and ultimately influence usage leading to hand hygiene compliance [27,28,29]. The overall result showed that gels and foams are more widely accepted compared to liquid, especially in terms of handleability, though the latter left a high clean feeling and took a shorter time to dry [30].

United States Food and Drug Administration (USFDA) has given the list of eligible antiseptic agents used in the non-prescription (also known as over-the-counter or OTC) and listed in Table 2. This list is highly useful in selecting appropriate antiseptic active ingredients intended for use by health care professionals in a hospital setting or other health care situations outside the hospital [31]. Recently, the United States Pharmacopeia (USP) Compounding Expert Committee (CMP EC) recommends the three formulations for compounding alcohol-based hand sanitizers for use during shortages associated with the COVID-19 pandemic and listed in Table 3 [32].

ABHS in the form of a spray which trigger stream aerosol solution allows direct contact of the alcohol solution with the target surface. However, there are several limitations associated with the sprays, including overspray, breathed by patients and flammability. Ready-to-use alcohol “Hand Sanitizing Wipes (HSW)” is a pre-wetted towelette containing disinfectants, antiseptics, surfactants, etc. in a sealed package ready for use in topical disinfection. The advantage of HSW is eliminating the possible contaminations and transfer of pathogen due to towelettes reuse. However, the longer storage time could increase the probability of losing antimicrobial/viricidal activity due to the possible binding of active ingredients onto the towelettes or by the degradation of the active ingredient [33]. 

### 3.2. Alcohol and Soaps

**Figure 3:**
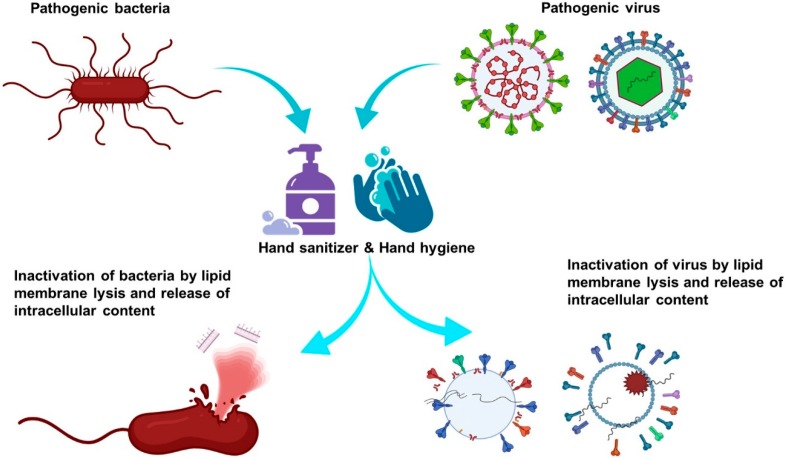
Illustration of alcohols antiviral mechanism. (Acknowledgement: The illustration was designed using BioRender scientific illustration program).

Keeping hands clean is a fundamental and essential step to avoid getting sick while limiting the transmission of germs to others. CDC recommends handwashing with soap and water whenever possible as it remarkably reduces the amount of all types of microbes and dirt on the skin surface [15,34]. Both the soaps and alcohol-based sanitizers work by dissolving the lipid membranes of microbes, thereby inactivating them (Figure 3). Thus, the sanitizer serves as an alternative when the soap and water are not readily available. The suggested minimum alcohol content of 60% is needed for it to exert the microbicidal effect. As compared to soap, alcohol-based sanitizers do not eliminate all types of germs, including norovirus and *Clostridium difficile*, the common pathogens that can cause diarrhoea [35,36]. While most people prefer to use sanitizers as they come in handy, and assume that the sanitizers may not be as effective as the soap at killing germs, this is because people may not use a sufficient amount of sanitizers to clean the hands [37,38]. The liquid may evaporate before it is evenly rubbed all over the hands, therefore compromising the efficacy of the sanitizers [37,39]. Also, the sanitizer may not work well when the hands are grossly dirty or contaminated with harmful chemicals [40].

Although hand sanitizers may be less effective than soaps in some situations, it is undeniable that they are the preferred form of hand hygiene in healthcare settings. The use of alcohol-based sanitizer may improve the compliance of healthcare workers to hand hygiene practices as they are easily accessible and take less time to use. Around 2.5–3 mL of liquid (equivalent to two pumps from a dispenser) is deposited on the palm and rubbed all over the surfaces of both hands for 25–30 s to maximize the efficacy of the sanitizer [41].

### 3.3. Pharmaceutical Ingredients and Their Function

ABHS contains either ethanol, isopropanol, or n-propanol. A concentration of 60%–95% of alcohol by volume is said to exhibit optimum bactericidal activity [42,43]. The antimicrobial effect of alcohols is attributed to their ability to dissolve the lipid membranes and denature the proteins of microbes. Alcohols have broad-spectrum antimicrobial activity against most vegetative forms of bacteria (including *Mycobacterium tuberculosis*), fungi, and enveloped viruses (human immunodeficiency virus [HIV] and herpes simplex virus). However, they are ineffective against bacterial spores that are found most commonly in raw materials. The addition of hydrogen peroxide (3%) may be a solution to this issue, but handling with caution during production is required due to its corrosive nature [41]. 

For alcohol-free products, various antiseptics have substituted alcohol as the main active ingredient. The mechanism of action of alcohols and non-alcohol compounds have been summarized in Table 4.

#### 3.3.1. Chlorhexidine

Similar to alcohol, chlorhexidine works by disrupting the arrangement of cytoplasmic membranes, thereby leading to precipitation of cell contents [44]. It is most effective against Gram-positive bacteria and has modest activity against a Gram-negative bacteria, as well as enveloped viruses [44,45]. As chlorhexidine is cationic, it is advisable to avoid using chlorhexidine-containing products with natural soaps and hand creams that contain anionic emulsifying agents as they may cause inactivation or precipitation of chlorhexidine, thus reducing its efficacy [44,45,46]. Chlorhexidine gluconate 0.12% is likely to have antiviral activity against the coronavirus as it does against other enveloped viruses [47].

#### 3.3.2. Chloroxylenol

Chloroxylenol is a common agent as a preservative in cosmetics or as an antimicrobial agent in soap. The antimicrobial effect of chloroxylenol is attributable to its ability to deactivate enzyme systems and alter cell wall synthesis in microbes. It is good at killing bacteria and enveloped viruses but less active against *Pseudomonas aeruginosa* [48,49].

#### 3.3.3. Iodine/Iodophors

Iodine was once an effective antiseptic used for skin disinfection. It can penetrate the microbial cell wall and form complexes with amino acids or unsaturated fatty acids to impair the synthesis of cellular components. Nonetheless, due to its potential to cause skin irritation and discoloration, iodophors have come into play to replace iodine as the active ingredient in antiseptics. The FDA has not cleared any liquid chemical sterilant or high-level disinfectants with iodophors as the main active ingredient [50].

Iodophors are a combination of either iodine, iodide or triiodide, and a high molecular weight polymer carrier such as polyvinyl pyrrolidone. This carrier is responsible for improving the solubility of iodine, enhancing the sustained release of iodine, and minimizing skin irritation [51]. The degree of antimicrobial activity determined based on the amount of free iodine present in the structure. Having said so, formulations with lower iodophor concentration may have significant antimicrobial activity as well because the amount of free iodine tends to increase after dilution [52].

Both iodine and iodophors exhibit germicidal activity against a Gram-positive, Gram-negative, and spore-forming bacteria, as well as various fungi and viruses [53,54,55]. However, the concentration of iodophors used in antiseptics (e.g., povidone-iodine 5%–10%) is usually insufficient to achieve sporicidal action. Furthermore, the nasal povidone-iodine formulation has shown acceptable tolerability and favorable risk/benefit profile to help mitigate the perioperative spread of COVID-19 in patient decolonization [56].

#### 3.3.4. Quaternary Ammonium Compounds

Quaternary ammonium compounds are composed of four alkyl groups connected to a nitrogen atom in the centre. The typical examples include benzalkonium chloride, benzethonium chloride, and cetyl peridium chloride. They act by adsorbing to the cytoplasmic membrane, thus causing leakage of the constituents. They are more active against Gram-positive bacteria and lipophilic viruses. The activity against fungi, mycobacteria, and Gram-negative bacilli is comparatively weak [15].

#### 3.3.5. Triclosan

At low concentration, triclosan is bacteriostatic due to its harmful effects to bacterial enzymes responsible for the composition of fatty acid from cells wall and membranes. At high concentrations, triclosan disrupts the bacteria membrane, leading it to death [8,57,58]. It has good activity against Gram-positive bacteria, including methicillin-resistant *Staphylococcus aureus*, *Candida* spp. and mycobacteria. The efficacy of triclosan may be affected by pH, use of emollients, and the ionic nature of certain skin formulations [58].

A lot of sanitizers also include humectant, for instance, glycerine, in the formulation to reduce the incidence of dry skin associated with the use of alcohol-based products as the alcohol can strip away sebum that helps to keep the skin moist. Though fragrance and colorant added to improve the aesthetics, it is generally not recommended to do so due to the risk of allergic reactions [41,43].

### 3.4. Physiology of Hand Skin

The skin is composed of three main layers: a superficial epidermis (50–100 μm), a middle dermis (≈2 mm), and an innermost hypodermis (1–2 mm). It constitutes the first line of defence against invading microorganisms while providing protection against mechanical impacts and preventing excessive loss of water from the body.

The vital barrier function of the skin resides primarily in the uppermost epidermal layer, the stratum corneum (SC). The SC contains layers of corneocytes that are terminally differentiated from keratinocytes that make up the basal layer of epidermis [15,59]. The adjacent corneocytes are interconnected by membrane junctions called corneodesmosomes to enhance the cohesion of the SC [60]. The lipids that are derived from the exocytosis of lamellar bodies during terminal differentiation of keratinocytes will fill up the intercellular spaces between the corneocytes, and they play a role in maintaining the cutaneous barrier function [61]. The layer underneath the SC is known as the keratinized stratified epidermis. It consists of melanocytes that produce melanin, a skin pigment that provides skin with its color and protects the skin from ultraviolet radiation. Apart from that, Langerhan’s cells, which are involved in the immune response and Merkel cells that are responsible for light touch sensation, can also be found within this layer [62,63].

Though the skin serves as a barrier that protects one against harmful microorganisms, it hosts a wide array of beneficial bacteria such as *Staphylococcus epidermis*, *Staphylococcus aureus*, *Micrococcus* spp., *Propionibacterium* spp. and *Corynebacterium* spp. [64,65]. These bacteria may help to prevent the colonization of pathogenic microbes by either competing with them for nutrients or stimulating the skin’s defence system. Under normal circumstances, they exhibit low pathogenicity. However, when the skin flora distribution is disrupted, for example, due to the long-term use of topical antibiotics or frequent hand washing, they may become virulent [66,67]. To reduce the incidence of infection, the microbiota balance is restored and maintained through constant skin regeneration. The whole process takes about 28 days, starting from the mitotic division of basal epithelium to desquamation. When the dead keratinocytes in the SC are sloughed off, it takes away the microbes that colonized the skin surface. This continuous process significantly limits the invasion of bacteria while achieving a balanced growth among the microbial populations.

### 3.5. Efficacy of Alcohol-Based Hand Sanitizer against the Coronavirus

The virus SARS-CoV-2 is termed due to of its genome sequence similarity to SARS Coronavirus (SARS-CoV) [68,69]. The CoVs belong to the same genus *Beta coronavirus*, sharing similar morphology in the form of enveloped, positive single-stranded RNA viruses [70,71]. These viruses can be deactivated by certain lipid solvents such as ethanol, ether (75%), chlorine-containing disinfectants, and chloroform, except chlorhexidine [70]. Ethyl alcohol, at concentrations of 60%–80%, is a potent viricidal agent inactivating all the lipophilic viruses (e.g., influenza, herpes and vaccinia virus) and many hydrophilic viruses (e.g., adenovirus, enterovirus, rhinovirus, and rotaviruses but not hepatitis A virus (HAV) or poliovirus) [32].

The 2015 WHO Model List of Essential recommended ethanol at 80% (v/v) and isopropyl alcohol at 75% (*v*/*v*) under the category ‘Disinfectant: Alcohol-based hand rub’ [72]. Ethanol (60%–85%) appears to be the most effective against viruses compared to isopropanol (60%–80%) and n-propanol (60%–80%) [23]. The study conducted with WHO-recommended alcohol-based formulations demonstrated a strong virucidal effect against emerging pathogens, including ZIKV, EBOV, SARS-CoV, and MERS-CoV [73]. Another study conducted in Germany found that the ethanol in the concentration of 42.6% (*w*/*w*) was able to destroy SARS coronavirus and MERS coronavirus within 30 s [74]. The efficacy of various alcohol-based sanitizers at different concentrations was also investigated in several studies, as shown in Table 5.

### 3.6. The Adverse Effects of Alcohol-Based Sanitizer or Handwashing Soaps

The most commonly reported skin reactions with the use of ABHS are irritant contact dermatitis (ICD) and allergic contact dermatitis (ACD) [76,77]. The symptoms of ICD can range from mild to debilitating with manifestations like dryness, pruritus, erythema and bleeding, if severe. As for ACD, the symptoms can either be mild and localized or severe and generalized, with most severe forms of ACD being manifested as respiratory distress or other anaphylactic symptoms [78,79]. Sometimes, it may be difficult to distinguish between ICD and ACD due to the overlap and similarities of symptoms.

Hand hygiene products such as sanitizer and soaps can be damaging to the skin through several mechanisms: denaturation of the stratum corneum proteins, alteration of intercellular lipids, decrease in corneocyte cohesion and reduction of stratum corneum water-binding capacity [80,81]. The biggest concern is the depletion of the lipid barrier, especially with repeated exposure to lipid-emulsifying detergents and lipid-dissolving alcohols as it may penetrate deeper into the skin layers and change the skin flora, resulting in more frequent colonization by bacteria [82,83,84]. In order of decreasing frequency of ICD including handwashing soaps are iodophors, chlorhexidine, chloroxylenol, triclosan and alcohol-based products. Among the alcohol-based formulations, ethanol has the least skin-irritant property compared to n-propanol and isopropanol [21]. There are, however, other contributing factors that increase the risk of ICDs such as lack of use of supplementary emollients, friction due to wearing and removal of gloves and low relative humidity [85,86,87]. ABHS also has a drying effect on hands which can further cause the skin to crack or peel [88,89,90].

On the other hand, ACD is caused by allergic reactions towards certain agents in the formulations such as iodophors, chlorhexidine, triclosan, chloroxylenol and alcohols [91]. Individuals with allergic reactions to alcohol-based preparations may have true allergy to alcohol or allergy to impurity, aldehyde metabolite or other excipients like fragrances, benzyl alcohol, parabens or benzalkonium chloride [29,92,93].

### 3.7. Recommendations to Minimize the Cutaneous Adverse Effects

The adverse effects caused by sanitizer or handwashing soaps can be easily prevented by identifying the trigger and countered with appropriate measures using one or a combination of following methods: selecting products with a less irritating agent, moisturizing skin after hand sanitation and avoiding habits that may cause or aggravate skin irritation [29,41,93,94].

When frequent hand cleansing is expected, for instance, among healthcare workers, it is preferable to select products that have a good balance between effectiveness, safety and compatibility with all skin types. The concerns about drying and irritant effects of alcohol or certain antiseptic soaps may hinder the acceptance and ultimate use of these preparations [52]. Hence, to reduce this problem, ABHS containing humectants or emollients can be used instead [95]. In recent years, novel water-based antiseptic lotions are also being studied such as that using benzethonium chloride, which not only addresses the issue regarding cutaneous adverse effects but also broadens the efficacy against viruses and tackles concerns about flammability associated with conventional ABHS [76].

Temperature and humidity are considered as significant contributors to the risk factors of dermatitis. The retention of skin moisture is longer in tropical countries and places with higher relative humidity compared to cold, dry environments [96]. This aspect calls for a varying need of emollients concerning respective environmental conditions and climates according to geographical locations. Some individuals, such as the elderly and healthcare workers who often wear occlusive gloves, are more prone to dry skin. Therefore, it is a good practice for these high-risk individuals to use moisturizers containing humectants, fats or oils to enhance skin moisture and improve skin barrier function [96].

### 3.8. Hand Hygiene Recommendations from CDC (USA), WHO and Malaysia Regulations

Proper hand hygiene by washing hands or using alcohol-based sanitizer is one of the most critical measures to prevent direct or indirect transmission of the COVID-19 as it reduces the number of the viable SARS-CoV-2 virus on contaminated hands. There are five instances that call for hand hygiene: before and after having direct contact with patients, before handling invasive devices for patient care, after exposure to body fluids or excretions, after contact with objects including medical appliances within proximity of the patient, and before starting any aseptic task [96].

The CDC recommends washing hands with soap and water whenever possible because handwashing reduces the amounts of all types of germs and chemicals on hands [97]. If soap and water are not available, using a hand sanitizer with a final concentration of at least 60% ethanol or 70% isopropyl alcohol inactivates viruses that are genetically related to, and with similar physical properties as, the COVID-19.

The action of handwashing can mechanically remove the microorganisms, but the removal of resident pathogens is more effective when hands are washed with preparations containing anti-microbial agents [96]. According to the Policies and Procedures on CDC, WHO and the Infection Control by Ministry of Health Malaysia, the recommended duration for the entire handwash procedure spans between 40 to 60 s using the standard 7-step technique.

Comparatively, sanitizer containing at least 60% alcohol is more effective in destroying the microorganisms than handwashing with anti-microbial soaps due to their ability to inactivate and destroy the microbes [96]. However, it should be noted that the ABHS may not be as effective if the hands are visibly soiled, dirty or greasy, so handwashing with soap and water is preferred under these circumstances. The duration to rub sanitizer all over the hand surfaces is approximately 20 to 30 s [96].

## 4. Conclusions

Proper hand hygiene is one of the essential infection control strategies as it can undeniably lower the likelihood of direct or indirect transmissions of microorganisms. The use of ABHS is becoming more common because of their rapid action and efficiency in killing microorganisms, mainly when handwashing using soap and water is not practical or convenient. There are, however, some situations in which handwashing is preferred as ABHS are less effective when the hands are visibly dirty or stained and cannot cover certain kinds of pathogens. It is vital to select ABHS with the appropriate amount of alcohol and practice the correct hand hygiene technique when cleaning hands to ensure all the microorganisms are effectively killed.

## Figures and Tables

**Table 1:** Chemical classification of commonly used disinfectants in hand sanitizer and their mechanism of antimicrobial action.

Chemical Group	Examples	Mechanism of Action
Alcohol	Ethanol (C_2_H_6_O) Iso-propanol (C_3_H_8_O)	Denaturation of proteins in the plasma membrane
Chlorine compounds	Hypochlorites (ClO^−^) (e.g., Sodium hypochlorite) Chlorine dioxide (ClO2) Chloramine-t trihydrate (C7H7ClNNaO2S)	Halogenation/oxidation of cellular proteins
Iodine compounds	Povidone-iodine (polyvinylpyrrolidone with iodine)	Iodine can easily penetrate through the cell membranes of pathogens Followed by attacking vital proteins, nucleotides and fatty acids of cell
Quaternary ammonium compounds	Benzalkonium chlorides, including alkyl dimethyl benzyl ammonium chloride (C_22_H_40_N^+^), Benzyl dimethyl octyl ammonium Chloride (C_17_H_30_ClN), Didecyl dimethyl ammonium chloride (C_22_H_48_ClN).	Lower surface tension Inactivate enzymes Degrade cell-proteins
Peroxygens	Hydrogen peroxide (H_2_O_2_) Peracetic acid (PAA) (C_2_H_4_O3)	Free-radical oxidation of essential cell components
(Bis) phenols	Triclosan	Penetrate cytoplasmic bilayer
Biguanide	Chlorhexidine	Ionic interaction Disrupt cell membrane

**Table 2:** List of hand antiseptic ingredients approved by the Food and Drug Administration (FDA) used in healthcare and over the counter (OTC) [30].

Active Ingredient	Patient Antiseptic Skin Preparations	Healthcare Personal Hand Wash	Healthcare Personal Hand Rub	Surgical Hand Scrub	Surgical Hand Scrub
Alcohol 60%–95%	Y	N	Y	N	Y
Benzalkonium chloride	Y	Y	Y	Y	N
Benzethonium chloride	Y	Y	N	Y	N
Chlorhexidine gluconate	N	N	N	N	N
Chloroxylenol	Y	Y	N	Y	N
Cloflucarban	Y	Y	N	Y	N
Fluorosalan	Y	Y	N	Y	N
Hexylresorcinol	Y	Y	N	Y	N
Iodine complex (ammonium ether sulfate and polyoxyethylene sorbitan monolaurate)	N	Y	N	Y	N
Iodine complex (phosphate ester of alkylaryloxy polyethylene glycol)	Y	Y	N	Y	N
Iodine tincture United States Pharmacopeia (USP)	Y	N	N	N	N
Iodine topical solution USP	Y	N	N	N	N
Nonylphenoxypoly (ethyleneoxy) ethanoliodine	Y	Y	N	Y	N
Poloxamer-iodine complex	Y	Y	N	Y	N
Povidone-iodine 5%–10%	Y	Y	N	Y	N
Undecoylium chloride iodine complex	Y	Y	N	Y	N
Isopropyl alcohol 70%–91.3%	Y	N	Y	N	Y
Mercufenol chloride	Y	N	N	N	N
Methylbenzethonium chloride	Y	Y	N	Y	N
Phenol (equal to or less than 1.5%)	Y	Y	N	Y	N
Phenol (greater than 1.5%)	Y	Y	N	Y	N
Secondary amyltricresols	Y	Y	N	Y	N
Sodium oxychlorosene	Y	Y	N	Y	N
Triclocarban	Y	Y	N	Y	N
Triclosan	Y	Y	N	Y	N
Combinations: Calomel, oxyquinoline benzoate, triethanolamine, and phenol derivative	Y	N	N	N	N
Combinations: Mercufenol chloride and secondary amyltricresols in 50% alcohol	Y	N	N	N	N
Combinations: Triple dye	Y	N	N	N	N

Y: Eligible for specified use; N: Ineligible for specified use.

**Table 3:** Formulation composition for compounding alcohol-based hand sanitizer (ABHS) based United States Pharmacopeia (USP) and World Health Organisation (WHO) recommendations [32].

Components	Formulation 1: Ethanol Antiseptic 80% Topical Solution	Formulation 2: Isopropyl Alcohol Antiseptic 75% Topical Solution	Formulation 3: Isopropyl Alcohol Antiseptic 75% Topical Solution
Ethanol 96%	833.3 mL	-	-
Isopropyl Alcohol 99%	-	757.6 mL	-
Isopropyl Alcohol 91%	-	-	824.2 mL
Hydrogen Peroxide 3%	41.7 mL	41.7 mL	41.7 mL
Glycerol 98%	14.5 mL	7.5 mL	7.5 mL
Water *, sufficient quantity to make	1000 mL	1000 mL	1000 mL

* Water may be distilled water, cold boiled potable water, reverse osmosis water, or filtered water.

**Table 4:** Mechanism of action of alcohols and non-alcohol compounds.

Ingredient	Function	Remarks
**Alcohol-Based**		
Alcohol	Denatures protein and lipid membrane of microorganisms.	Optimum concentration 60%–95%.
Hydrogen peroxide	Inactivates contaminating spores in the bulk solutions or excipients.	Concentration is as low as 3%. May fade the coloring agent Corrosive in nature
**Non-Alcohol Based**		
Chlorhexidine gluconate	Inhibits the growth of microorganisms on living tissues.	Good activity o Gram-positive bacteria o Enveloped viruses ^a^ Weak activity o Gram-negative bacteria o Fungi o Non-enveloped viruses ^b^
Chloroxylenol		Good activity o Gram-positive bacteria o Gram-negative o Enveloped viruses Weak activity o *Pseudomonas aeruginosa*
Iodine/Iodophors		Gram-positive bacteria Gram-negative bacteria Fungi Enveloped viruses Spore-forming bacteria ^c^
Quaternary ammonium compounds Benzalkonium chloride Benzethonium chloride Cetylpyridinium chloride		Good activity o Gram-positive bacteria o Enveloped viruses Weak activity o Gram-negative bacteria o Mycobacteria o Fungi
Triclosan		Good activity o Gram-positive bacteria o Mycobacteria o *Candida* spp. Weak activity o Filamentous fungi
**Excipients**		
Glycerol	Acts as a humectant that maintains the skin moisture.	A lower concentration is considered to reduce the stickiness of the formulation.
Essential oils	Antibacterial, antiviral, antimicrobial and antiseptic properties, Flavoring agent	
Xanthum gum, polyacrylic acid and polyethylene glycol	Thickening agents	To enhance the viscosity of products
Fragrance and colorant	Aesthetic Aesthetic Allows differentiation from other fluids.	May cause allergic reactions.

^a^ herpes simplex virus, influenza, HIV, cytomegalovirus; ^b^ enterovirus, rotavirus, adenovirus; ^c^ *Clostridium* spp., *Bacillus* spp.

**Table 5:** Efficacy of different types of alcohol-based sanitizers at various concentrations against severe acute respiratory syndrome (SARS) coronavirus.

Formulation	Concentration	Exposure Time (s)	Efficacy Against SARS CoV	Ref
45% propan-2-ol (*w*/*w*) 30% propan-1-ol (*w*/*w*) 0.2% mecetronium ethyl sulphate	Undiluted	30	RF: ≥4.25	[75]
80% ethanol (*w*/*w*)	Undiluted	30	RF: ≥4.25	
85% ethanol (*w*/*w*)	Undiluted	30	RF: ≥5.5	
95% ethanol (*w*/*w*)	Undiluted	30	RF: ≥5.5	
85% ethanol (*v*/*v*) 0.725% glycerol (*v*/*v*) 0.125% hydrogen peroxide (*v*/*v*)	20%	30	Log_10_ of viral infection: 7	[73]
	40%–80%	30	Log_10_ of viral infection: Undetectable level	
75% isopropanol (*w*/*w*) 0.725% glycerol (*v*/*v*) 0.125% hydrogen peroxide (*v*/*v*)	20%	30	Log_10_ of viral infection: 6.8	
	40%–80%	30	Log_10_ of viral infection: Undetectable level	

RF: Reduction factor (calculated as the difference in the quotient of control titration and after incubation of the virus with the disinfectant). Higher RF value indicates higher virus reduction potential. Log_10_ value of ≤1 is not significant or ineffective, log_10_ value of 1–2 is indicative/contributable effective, log_10_ value of 2–4 is moderately effective, and log_10_ value of ≥4 is highly effective. Undetectable level indicates a higher potential than is demonstrated.
